# PipeVal: light-weight extensible tool for file validation

**DOI:** 10.1093/bioinformatics/btae079

**Published:** 2024-02-10

**Authors:** Yash Patel, Arpi Beshlikyan, Madison Jordan, Gina Kim, Aaron Holmes, Takafumi N Yamaguchi, Paul C Boutros

**Affiliations:** Jonsson Comprehensive Cancer Center, University of California, Los Angeles, Los Angeles, CA 90095, United States; Institute for Precision Health, University of California, Los Angeles, Los Angeles, CA 90095, United States; Jonsson Comprehensive Cancer Center, University of California, Los Angeles, Los Angeles, CA 90095, United States; Institute for Precision Health, University of California, Los Angeles, Los Angeles, CA 90095, United States; Jonsson Comprehensive Cancer Center, University of California, Los Angeles, Los Angeles, CA 90095, United States; Institute for Precision Health, University of California, Los Angeles, Los Angeles, CA 90095, United States; Department of Human Genetics, University of California, Los Angeles, Los Angeles, CA 90095, United States; Jonsson Comprehensive Cancer Center, University of California, Los Angeles, Los Angeles, CA 90095, United States; Institute for Precision Health, University of California, Los Angeles, Los Angeles, CA 90095, United States; Jonsson Comprehensive Cancer Center, University of California, Los Angeles, Los Angeles, CA 90095, United States; Institute for Precision Health, University of California, Los Angeles, Los Angeles, CA 90095, United States; Jonsson Comprehensive Cancer Center, University of California, Los Angeles, Los Angeles, CA 90095, United States; Institute for Precision Health, University of California, Los Angeles, Los Angeles, CA 90095, United States; Department of Human Genetics, University of California, Los Angeles, Los Angeles, CA 90095, United States; Jonsson Comprehensive Cancer Center, University of California, Los Angeles, Los Angeles, CA 90095, United States; Institute for Precision Health, University of California, Los Angeles, Los Angeles, CA 90095, United States; Department of Human Genetics, University of California, Los Angeles, Los Angeles, CA 90095, United States; Department of Urology, University of California, Los Angeles, Los Angeles, CA 90095, United States; Broad Stem Cell Research Center, University of California, Los Angeles, Los Angeles, CA 90095, United States

## Abstract

**Motivation:**

The volume of biomedical data generated each year is growing exponentially as high-throughput molecular, imaging and mHealth technologies expand. This rise in data volume has contributed to an increasing reliance on and demand for computational methods, and consequently to increased attention to software quality and data integrity.

**Results:**

To simplify data verification in diverse data-processing pipelines, we created PipeVal, a light-weight, easy-to-use, extensible tool for file validation. It is open-source, easy to integrate with complex workflows, and modularized for extensibility for new file formats. PipeVal can be rapidly inserted into existing methods and pipelines to automatically validate and verify inputs and outputs. This can reduce wasted compute time attributed to file corruption or invalid file paths, and significantly improve the quality of data-intensive software.

**Availability and implementation:**

PipeVal is an open-source Python package under the GPLv2 license and it is freely available at https://github.com/uclahs-cds/package-PipeVal. The docker image is available at: https://github.com/uclahs-cds/package-PipeVal/pkgs/container/pipeval.

## 1 Introduction

Over the past decade, biomedical research has been transformed by the advent of high-throughput technologies that rapidly and affordably generate extremely large datasets. These have derived from a wide-range of technologies, ranging from next-generation sequencing, live-cell imaging, and mHealth (the practice of medicine and public health through mobile devices). Each of these high-throughput and scalable technologies has contributed to a dramatic, ongoing increase in data volume, coupled to increasing demand for processing tools and algorithms. Discovery research has grown to rely on the development and routine application of algorithms and software for post-processing, denoising, quality-control, feature-engineering, and other pre-processing steps. This has made the quality of software increasingly central to the innovation and execution of biomedical research (Dash *et al.* 2019, Cremin *et al.* 2022).

High-quality software development involves a set of practices widespread in some settings, but typically deprioritized in academic settings. This is especially true during the early phase of rapid algorithmic innovation, when good software engineering is often secondary to determining the optimal strategy for a new algorithm; utility is prioritized over implementation. The need for continued publication of novel work can then disincentivize refactoring and other software-quality improvements, except for the most widely-used tools (Koru *et al.* 2007, Silva *et al.* 2017). There are many prominent and well-known good software engineering practices, including test-driven development (Patel *et al.* 2024*Bioinformatics* in press), developer and user documentation, requirements management, and input/output validation.

The reasons why each of these is not routine vary, but the barriers to systematic input and output validation are straight-forward: implementing them can be cumbersome, time-consuming, and repetitive. Each function or file-type requires its own bespoke validation, increasing code-bloat and reducing readability. Creating these validations during development can slow down testing and feels cumbersome to developers; and yet, input and output validation is highly beneficial. Validation of inputs can reduce unnecessary compute cycles and reduce the risk of incorrect results. Validation of outputs can avoid unexpected results in downstream analyses and make software failures clear to end-users.

The lack of systematic input and output validation in many data-processing pipelines has been exacerbated by the growing popularity of workflow orchestration frameworks such as Snakemake and Nextflow (Köster and Rahmann 2012, Di Tommaso *et al.* 2017). These frameworks facilitate complex data engineering paths where up to dozens of programs can be executed in sequence and/or parallel to generate a diversity of outputs. However, file validation and error-checking are not intrinsic features of these frameworks, especially in the context of bioinformatics workflows involving a broad range of file formats. This creates an important need for a validation tool capable of handling a multitude of file types that can be plugged into any arbitrary data-processing workflow without requiring repeated wrapping of specific validation modules into individual workflows.

To fill this gap, we created PipeVal: a light-weight, easy-to-use, extensible tool for validating common file formats that reduces the friction and repetitiveness of bioinformatics input/output validation. It allows developers to rapidly validate formats including: FASTQ, SAM (Sequence Alignment/Map), BAM (Binary Sequence Alignment/Map), CRAM (Compressed Reference-oriented Alignment Map), ZIP, and VCF (Variant Call Format) (Li *et al.* 2009, Cock *et al.* 2010, Danecek *et al.* 2011, Cochrane *et al.* 2012). It has generic checksum generation and verification, and is trivially extensible to new file formats. It can be incorporated into existing pipelines with just a single line of code. By reducing barriers to file validation, PipeVal can help improve bioinformatics software quality.

## 2 Results

PipeVal is designed to be portable and easy to install on a range of systems. It is installable through the Python package manager pip, which handles installation of dependencies. It also supports cross-platform compatibility through an accompanying light-weight Docker image (581 MB) built to encapsulate dependencies and the utility itself for use on any system. The Docker image is particularly convenient for use in complex workflows, such as those using Snakemake, Nextflow, or other workflow orchestration frameworks (Merkel 2014, Di Tommaso *et al.* 2017, Mölder *et al.* 2021).

To maximize ease of implementation into existing workflows, the PipeVal interface is extremely simple, with the main command being pipeval, with subcommands for validation (pipeval validate) and for checksum generation (pipeval generate-checksum). The standard command to validate a file is simply pipeval validate file.extension. PipeVal automatically handles proper detection of the specific file-type based on extensions and customizes the validation performed on that basis.

**Figure 1.**
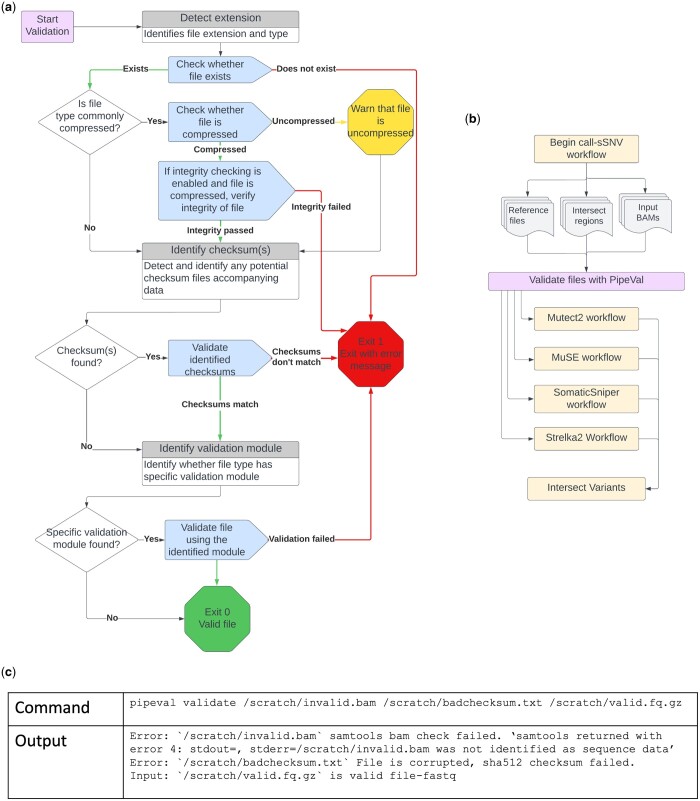
PipeVal validations and example command and output. (a) PipeVal validation workflow indicating the three common validations—existence, compression, and checksums—and the format-specific validations. (b) Example of integration of PipeVal into call-sSNV workflow. (c) Example command and output for invalid files, files with bad checksums, and valid files.

PipeVal begins by verifying that a file exists—this trivial check automatically handles a wide variety of path- and permission-related errors that occur routinely in computational biology (Fig. 1). It then considers whether the file is compressed (or potentially compressed) and it proceeds to verifying the compression and, if the integrity checking option is enabled, the integrity of the compressed file. Next, PipeVal automatically detects the presence of checksums in several formats (e.g. sha512, md5), and performs checksum verification. These validations are encapsulated within PipeVal and are easily integrable within workflows (Fig. 1).

These three validations—existence, compression, and checksums—are performed for all files. PipeVal then consults a library of modular file format-specific validation functions. Specific validation functions that exist from other works have been consolidated within PipeVal in order to support validation of multiple file types. As an example, the pysam module containing libraries for manipulating genomic data files such as BAM/SAM/CRAM is utilized for validation of those formats (Bonfield *et al.* 2021). The VCFtools suite, which includes a module for VCF file validation, is also leveraged within PipeVal (Danecek *et al.* 2011). If it is able to identify a validator for the type of a file, it then applies it. As one example, if PipeVal recognizes a BAM file, it will verify that the data contained is sequence data, verify that the header is properly formatted, and check for the presence of at least one read. PipeVal will also automatically detect and confirm the presence of an accompanying index file for BAM file. As a second example, when PipeVal recognizes a VCF file, it will check for properly formatted headers and for properly formatted variant calls.

PipeVal can validate multiple files in a single pass, and can generate checksums when they are not already present. With complex bioinformatics workflows involving many files, parallelization of validation is an important optimization consideration. PipeVal has been implemented with the option of validating multiple files in parallel by leveraging multi-threading. Extension to new file formats is modular: the automated file-type detection is linked to a mapping of validation modules to file types. The independence and integration of these modules enables quick addition of modules for new file types with a simple update to the file type-to-extension mapping and the file type-to-validation module mapping. PipeVal will also continue to optimize the validation process for large genomic files, by leveraging parallel processing techniques and specific file manipulation tools. Genomic files are often large and stored in compressed binary formats, which allows for the creation of index files to facilitate navigation. This property can therefore be leveraged to parallelize validation across multiple threads, with each one processing a subset of the file with access expedited through the index. Validation tools also may include options for quick validation aimed toward performing the basic checks. PipeVal encapsulates these options to support both fast, efficient validation, and in-depth, thorough validation using more resources depending on the workflow necessities.

With biomedical research becoming increasingly reliant on complex software workflows, software quality is becoming a key factor in innovation and replication. Standard good software engineering practices are often secondary in academic research. By greatly reducing the friction for standardized input/output validation, PipeVal can be easily incorporated into existing workflows to help verify data integrity. It can reduce the frustrating occurrence of corrupt files or incorrect file paths leading to wasted compute resources and in some cases incorrect results. PipeVal is freely available at: https://github.com/uclahs-cds/package-PipeVal. The docker image is available at: https://github.com/uclahs-cds/package-PipeVal/pkgs/container/pipeval.

## Data Availability

No new data were generated or analysed in support of this research.
